# Financial Security at Older Ages

**DOI:** 10.1093/geroni/igaa057.2393

**Published:** 2020-12-16

**Authors:** Stipica Mudrazija, Barbara Butrica

**Affiliations:** Urban Institute, Washington, District of Columbia, United States

## Abstract

As Americans have become increasingly indebted over time, understanding the factors influencing indebtedness is increasingly important and can help policymakers design policies that discourage debt, thus enhancing private retirement savings so that retirees become less dependent on Social Security benefits. The paper uses data from a major credit bureau of a nationally representative 2 percent random sample from more than 250 million consumer records to examine the financial health and indebtedness of older adults. The data cover the years 2010 through 2018 and follow the same consumers over time. They are supplemented with the zip-code level information from the American Community Survey on neighborhood characteristics, including racial and ethnic composition, education, median income, and poverty. Our preliminary findings suggest that, while the proportion of older adults in serious financial distress is not substantial, their indebtedness is increasing and the growth of unsecured debt (e.g., student debt) has been especially fast. Part of a symposium sponsored by the Economics of Aging Interest Group.

